# Different Doses of Intravenous Tissue-Type Plasminogen Activator for Acute Ischemic Stroke: A Network Meta-Analysis

**DOI:** 10.3389/fneur.2022.884267

**Published:** 2022-06-23

**Authors:** Bing-Hu Li, Jian-Hong Wang, Han Wang, Duo-Zi Wang, Shu Yang, Fu-Qiang Guo, Neng-Wei Yu

**Affiliations:** ^1^Department of Neurology, Sichuan Provincial People's Hospital, University of Electronic Science and Technology of China, Chengdu, China; ^2^Department of Outpatient, People's Liberation Army the General Hospital of Western Theater Command, Chengdu, China

**Keywords:** tissue-type plasminogen activator, ischemic stroke, network meta-analysis, intravenous thrombolysis, symptomatic intracranial hemorrhage

## Abstract

**Background:**

This study aims to assess the efficacy and safety of different doses of intravenous tissue-type plasminogen activator (tPA) for acute ischemic stroke (AIS) by adopting a network meta-analysis (NMA).

**Methods:**

Studies comparing different doses of tPA in AIS were identified by retrieving electronic databases. NMAs of outcome measures included favorable functional outcome with a modified Rankin scale score (mRS) of 0 or 1 at 3 months after treatment (3M-FF), the functional independence with a mRS of 0, 1, or 2 at 3 months (3M-FI), symptomatic intracranial hemorrhage (sICH) and 3-month all-cause mortality (3M-M). Symptomatic intracranial hemorrhage (sICH) and 3-month all-cause mortality (3M-M) were assessed. Probability-based ranking and surface under cumulative ranking (SUCRA) were performed to identify the best dose of tPA. Inconsistency was evaluated by node-splitting analysis and a loop-specific approach. Publication bias was analyzed by funnel plots.

**Results:**

A total of 14 studies were included in the quantitative synthesis. The NMA results revealed no difference among low (<0.7 mg/kg), moderate (0.8 mg/kg), and standard (0.9 mg/kg) doses of tPA with regard to efficacy and safety. The SUCRAs of 3M-FF and 3M-FI showed that the standard dose ranked first, the moderate dose ranked second, and the low dose ranked third. The SUCRA of sICH showed that the standard dose ranked first (78.1%), the low dose ranked second (61.0%), and the moderate dose ranked third (11.0%). The SUCRAs of 3-month mortality showed that the standard dose ranked first (73.2%), the moderate dose ranked second (40.8%), and the low dose ranked third (36.1%). No significant inconsistency was shown by node-splitting analysis and no publication bias was shown in funnel plots.

**Conclusion:**

Lower dose tPA was comparable to the standard dose with regard to efficacy and safety. Based on the SUCRA results and American Heart Association/American Stroke Association (AHA/ASA) guidelines, the standard dose was still the optimal selection for AIS.

## Introduction

Globally, stroke remains the second-leading cause of death. Prevalent cases of stroke were estimated to be 101 million in 2019, 62.4% of which were ischemic stroke (1). Until now, tissue plasminogen activator (tPA) is the only treatment approved by the US Food and Drug Administration (FDA) serving as the first line of treatment for acute ischemic stroke (AIS) (2). However, due to the high cost, narrow therapeutic time window, and risk of intracranial hemorrhage, tPA is clinically limited. Therefore, it is urgent to find ways to reduce the medical burden and risk of intracranial hemorrhage. Under this context, several studies (3–5) were performed to explore the efficacy and safety of a lower dose of tPA for AIS in Japan, which demonstrated that the efficacy and safety of low dose (0.6 mg/kg) tPA was comparable to a dose of 0.9 mg/kg from published data.

Thereafter, numerous studies compared the efficacy and safety of different doses of tPA for AIS, which remain inconclusive. A previous meta-analysis aimed at analyzing whether low dose tPA can effectively reduce symptomatic intracranial hemorrhage (sICH) and has the same efficacy as standard dose tPA showed that low dose tPA was comparable to standard-dose tPA in terms of efficacy and safety in Asian patients with AIS (6). However, in this meta-analysis, the low dose was defined as <0.75 mg/kg, and the standard dose was defined as >0.75 mg/kg. In addition, five new studies (7–11) were reported after this meta-analysis. Moreover, traditional meta-analysis is difficult to use to assess the effects of two or more interventions. By contrast, network meta-analysis (NMA) can make comparisons of all the interventions and can also provide information on which is the best treatment by ranking analysis. Thus, in this study, we will perform an NMA by combining all studies concerning different doses of tPA for AIS to compare their efficacy and safety.

## Methods

### Literature Search

The presentation of this study followed the Preferred Reporting Items for Systematic Reviews and Meta-Analyses (PRISMA) reporting guideline (12). A systematic search of PubMed, Embase, and Web of Science (last search was updated on 4 January 2022) was performed with a combination of the following keywords: (stroke/cerebral infarction/cerebral ischemia) with (alteplase OR tissue plasminogen activator OR intravenous thrombolysis OR rtPA) and low dose (Supplementary Table 1). The language was restricted to English. We manually searched the references of articles retrieved. When the same patient population was used in several publications, only the complete or largest study was included. Two investigators screened each of the titles, abstracts, and full texts to determine inclusion independently. The results were compared and disagreements were resolved by consensus.

### Selection Criteria

The inclusion criteria were as follows: (1) studies with retrospective and prospective cohort design; (2) studies should report functional outcome (with modified Rankin scale score [mRS] assessment) at 3 months after symptom onset, incidence of mortality, and sICH; (3) studies with full-text articles; and (4) studies comparing the effect of different doses of tPA in AIS.

The exclusion criteria were as follows: (1) non-control study or placebo-control studies; (2) studies sharing the same patient population; (3) studies with data that could not be extracted or converted into valid data.

### Data Extraction

Information was carefully extracted from all included publications independently by the two authors according to the inclusion criteria listed above. Disagreement was resolved by consensus or discussion with a third reviewer. The following data were collected from each study: first author's name, publication date, country, number of patients, the dose of tPA, the incidence rate of favorable functional outcome at 3 months after treatment (3M-FF), functional independence at 3 months (3M-FI), sICH, and 3-month all cause-mortality (3M-M), respectively.

### Data Synthesis

The doses of tPA were classified as low (<0.7 mg/kg), moderate (0.8 mg/kg), and standard (0.9 mg/kg).

### Outcome Measures

The efficacy outcomes included the proportion of patients achieving an mRS of 0 or 1 at 3M-FF and an mRS of 0, 1, or 2 at 3M-FI. The safety outcome included the incidence rate of sICH and 3M-M. The sICH is defined by the European Cooperative Acute Stroke Study (ECASS) criteria (13). If the sICH data defined by ECASS criteria were not available, the incidence rate of sICH defined by National Institute of Neurological Disorders and Stroke (NINDS) (14) was applied.

### Assessment of Risk of Bias

Risk of bias was assessed using the Cochrane Collaboration Tool. Judgment as “low,” “unclear,” or “high” risk of bias was provided in each of the domains for each study.

### Statistical Analyses

A network meta-analysis was carried out using STATA version 15.0 based on the Bayesian framework model. The corresponding odds ratios (ORs) with 95% CIs were calculated. Rank plots based on probabilities and the surface under cumulative ranking (SUCRA) for different outcomes were performed to identify the best treatment. Inconsistency was evaluated by node-splitting analysis and a loop-specific approach. Publication bias was analyzed by funnel plots.

## Results

### Characteristics of the Study

**Figure 1:**
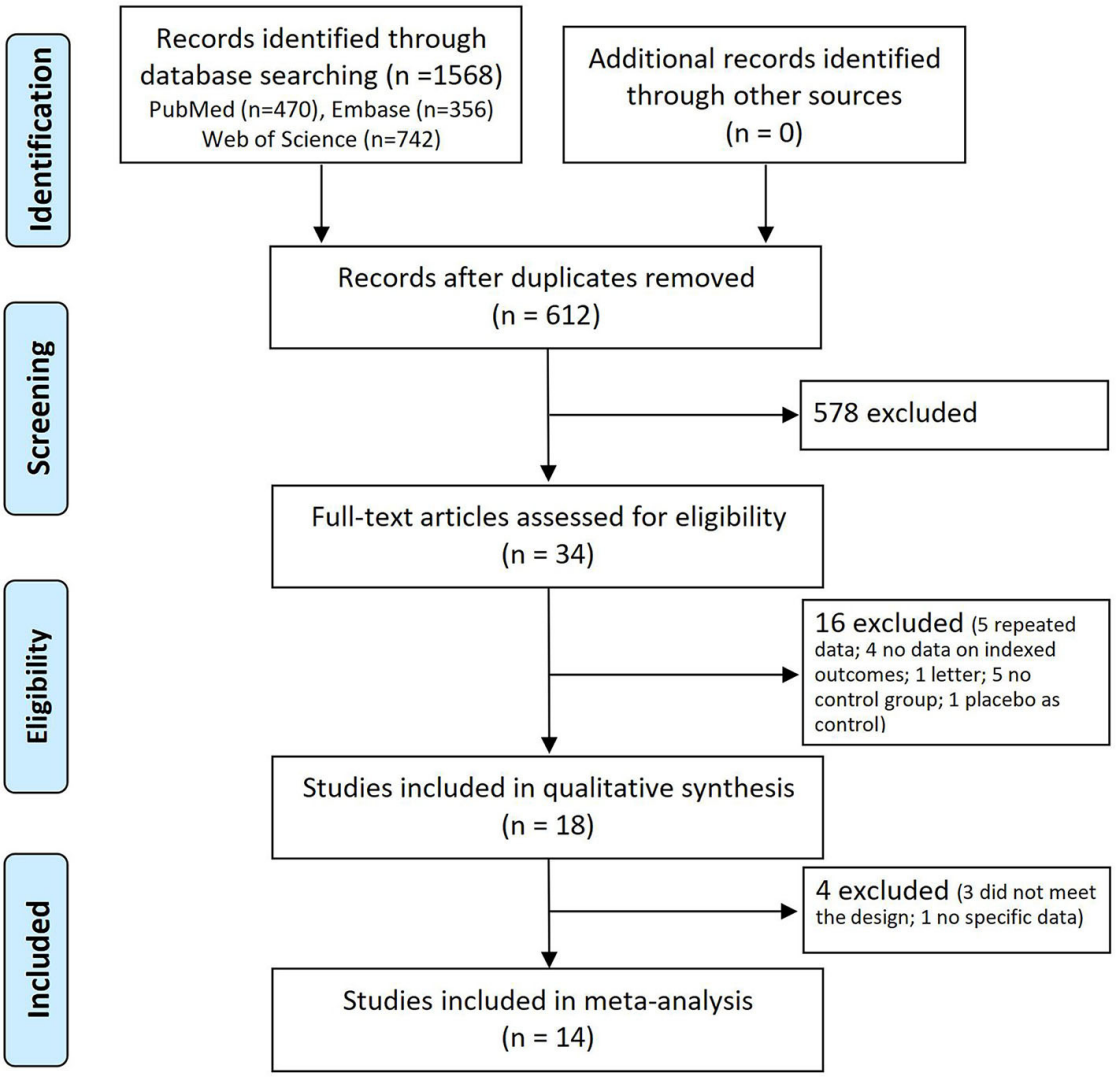
Flow diagram of the study selection process.

The study selection process is detailed in Figure 1. There were 1,568 potentially relevant articles identified after the search. After screening titles and abstracts, a total of 34 studies were included for full-text article assessment. Five studies were non-control studies (3–5, 15, 16). One study was a placebo-control study (17). Two studies reported the same cohort (18, 19), and the larger study (19) was included. Four studies reported the same cohort (20–23), and the largest study (20) was included. One study was an individual patient data pooling study from six Asian countries (China, Japan, Philippines, Singapore, South Korea, and Taiwan) (24). One study was a letter to the editor (25). Four studies provided no data on indexed outcomes (26–29).

Finally, 18 studies were included in the qualitative synthesis (7–11, 19, 20, 30–40). The doses of three studies (30, 34, 39) did not meet the design of the present study and one study (40) without specific data was excluded from the final quantitative analysis. Fourteen studies were included in the quantitative synthesis (7–11, 19, 20, 31–33, 35–38). Table 1 summarizes the characteristics of the 14 included studies. In brief, these studies were reported between 2010 and 2021. The majority (10/14) were two-arm studies; four studies had three arms. Seven studies were retrospective cohort studies, one study was a randomized controlled trial, and six studies were prospective cohort studies. Eight studies were from China, one study was from Singapore, one study was from Korea, one study was from the Czech Republic, one study was from Srpska, one study was from Egypt, and one study was worldwide. The risk of bias of included studies in this NMA was generally low to unclear. Details about the risk of bias assessment are graphically summarized in Supplementary Figure 1.

**Table 1:** Characteristics of the included studies.

**References**	**Country**	**Study design**	**Intervention**	**N**	**3M-FF**	**3M-FI**	**sICH**	**3M-M**
Sharma et al. (31)	Singapore	Retrospective cohort	0.67 mg/kg	48	17/48		7/48	5/48
			0.9 mg/kg	82	48/82		1/82	11/82
Zhou et al. (32)	China	Retrospective cohort	0.6–0.7 mg/kg	23	8/23	12/23	1/23	4/23
			0.8 mg/kg	31	12/31	16/31	1/31	5/31
			0.9 mg/kg	51	26/51	33/51	2/51	6/51
Chen et al. (33))	China	Retrospective cohort	0.7 mg/kg	105	39/95	50/95	5/105*	8/105
			0.9 mg/kg	156	56/146	79/146	4/156*	9/156
Aulicky et al. (35)	Czech	Retrospective cohort	0.78 ± 0.06 mg/kg	62	31/62		4/62	13/62
			0.9 ± 0.03 mg/kg	171	87/171		8/171	21/171
Chao et al. (19)	China	Prospective cohort	0.6–0.7 mg/kg	380	100/302	147/302	16/380	33/380
			0.8 mg/kg	202	46/171	76/171	11/202	18/202
			0.9 mg/kg	422	124/367	173/367	21/422	35/422
Liao et al. (36)	China	Prospective cohort	0.64 mg/kg	75	31/74	42/74	0/75	14/74
			0.79 mg/kg	131	61/127	69/127	11/131	11/127
			0.9 mg/kg	678	358/665	429/665	21/678	49/666
Kim et al. (37)	Korea	Prospective cohort	0.6 mg/kg	450	146/450	205/450	38/450	57/450
			0.9 mg/kg	1,076	380/1,076	526/1,076	69/1,076	151/1,076
Anderson et al. (20)	Worldwide	Randomized controlled trial	0.6 mg/kg	1,654	752/1,607	1,002/1,607	98/1,607*	140/1,607
			0.9 mg/kg	1,643	782/1,599	1,007/1,599	131/1,599*	170/1,599
Yang et al. (38)	China	Retrospective cohort	0.6 mg/kg	46	34/46		2/46	
			0.9 mg/kg	62	44/62		3/62	
Ong et al. (8)	China	Retrospective cohort	0.6–0.7 mg/kg	130	40/130	48/130	6/130	7/130
			0.8 mg/kg	88	34/88	43/88	1/88	1/88
			0.9 mg/kg	56	13/56	17/56	3/56	1/56
Chao et al. (9)	China	Prospective cohort	0.6 mg/kg	108	15/108	24/108	7/108*	10/108
			0.9 mg/kg	141	32/141	49/141	6/141*	19/141
Liu et al. (7)	China	Prospective cohort	0.5–0.7 mg/kg	60	17/60	22/60	2/60	11/60
			0.85–0.95 mg/kg	494	209/494	259/494	20/494	66/494
Škrbić et al. (10)	Srpska	Retrospective cohort	0.6 mg/kg	45		24/45		0/45
			0.9 mg/kg	165		106/165		10/165
Salem et al. (11)	Egypt	Prospective cohort	0.6 mg/kg	40		27/40	0/40	3/40
			0.9 mg/kg	40		25/40	3/40	2/40

*N, number of patients; 3M-FF, 3-month favorable functional outcome (mRS of 0, 1); 3M-FI, 3-month functional independence (mRS of 0, 1, or 2); 3M-M, 3-month mortality; sICH, symptomatic intracranial hemorrhag*;

* *sICH was defined by NINDS*.

### Results of Network Meta-Analysis

**Figure 2:**
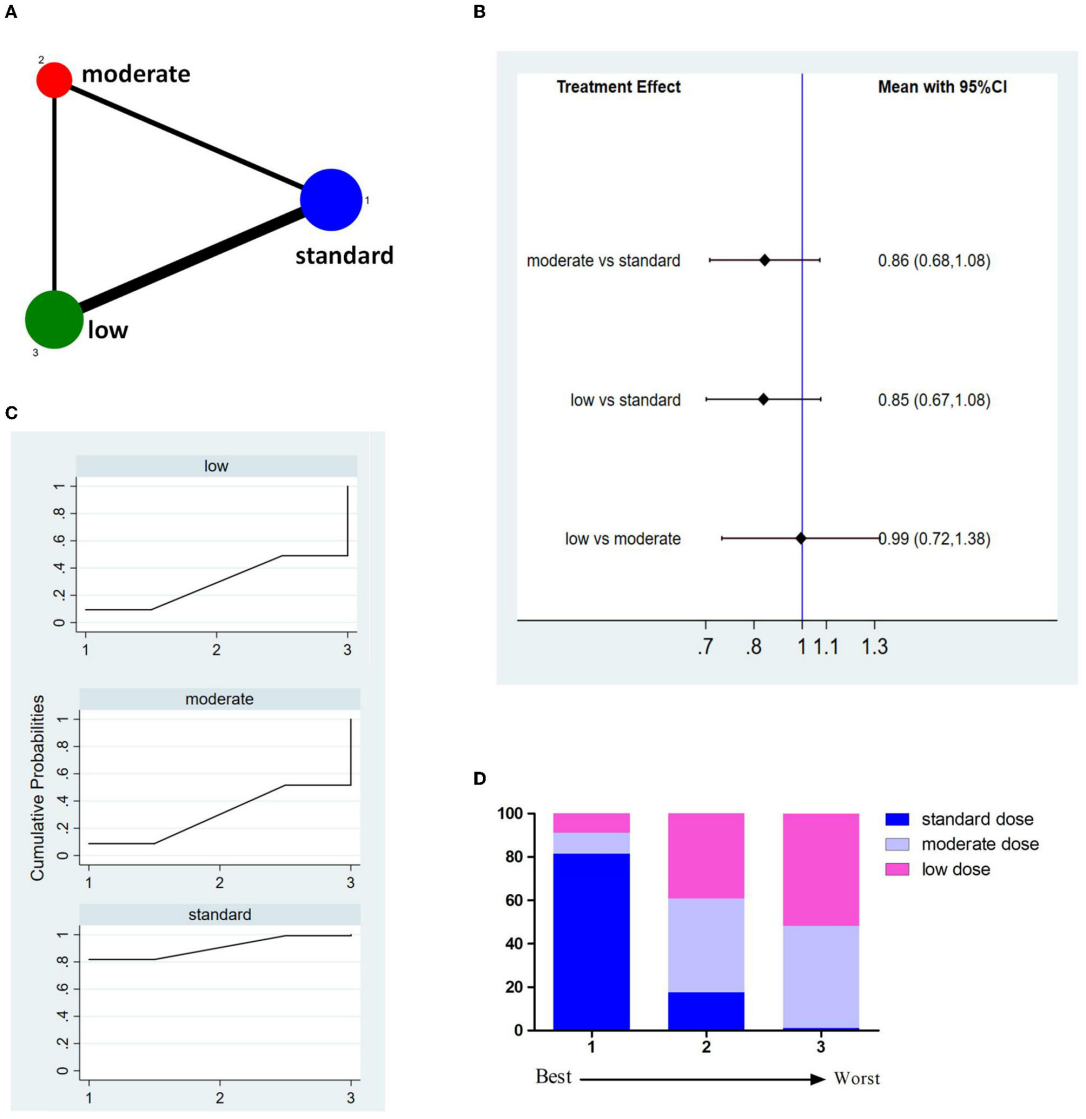
Results of favorable functional outcome at 3 months after treatment (3M-FF). **(A)** Network plots of eligible comparisons. The width of the lines represents the number of studies being compared, and the node size reflects the sample size. **(B)** The forest plot of network results. The black diamonds represent the combined ORs; OR > 1 indicates that the proportion of 3M-FF in the former group is greater than that in the latter group. **(C)** The cumulative ranking curve of 3M-FF. **(D)** The ranking of different doses of tPA is based on the cumulative probability plots. Ranking first means having the highest proportion of 3M-FF.

Twelve studies with 8,332 patients reported 3M-FF (Figure 2A). The pooled meta-analysis results (Figure 2B) showed no significant difference between different doses of tPA. The probability-based ranking result is shown in Figures 2C,D. Results of SUCRA showed that the standard dose ranked first (90.2%), the moderate dose ranked second (31.3%), and the low dose ranked third (28.5%). In this result, the first rank had the highest proportion of 3M-FF.

**Figure 3:**
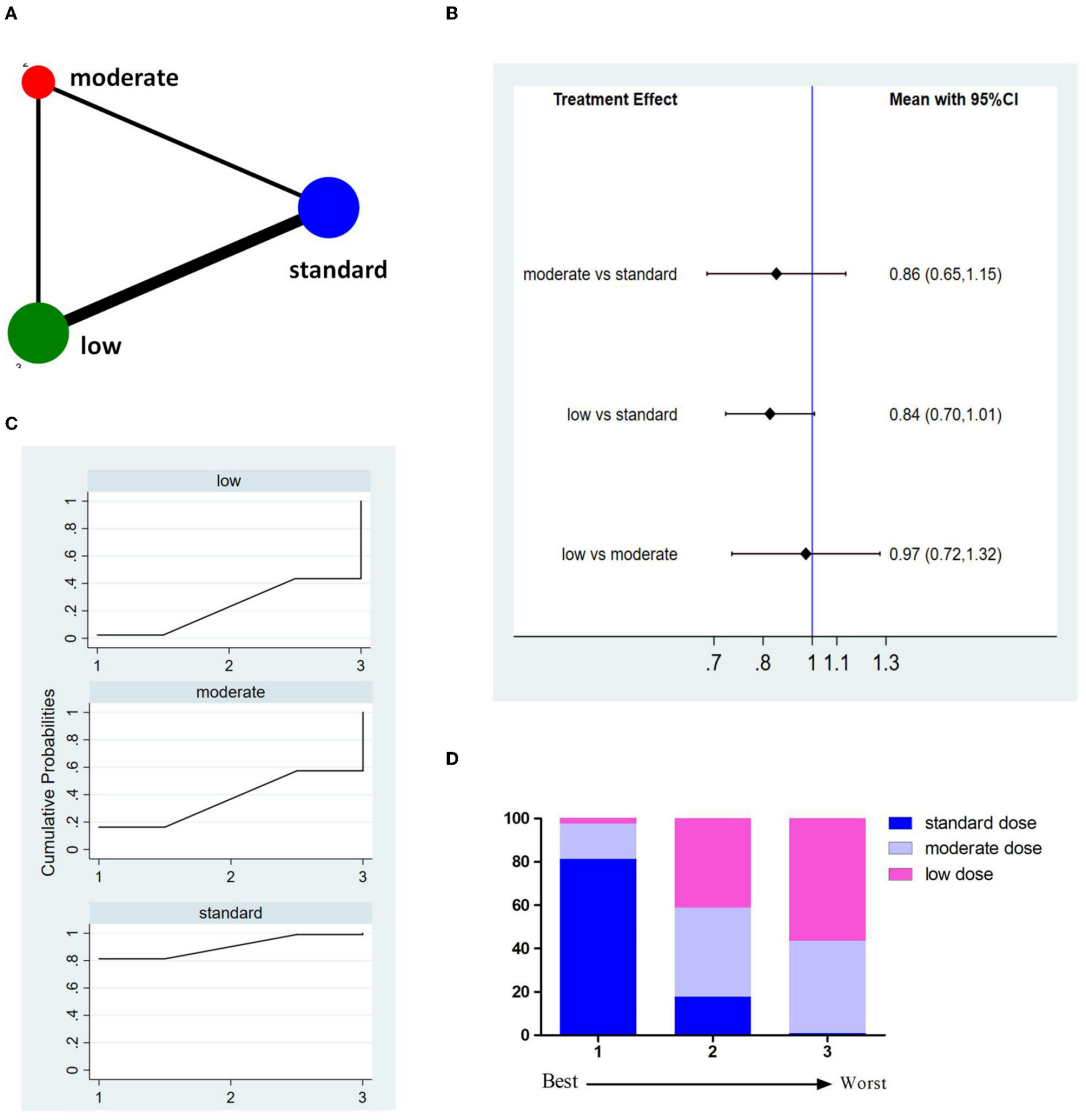
Results of functional independence at 3 months (3M-FI). **(A)** Network plots of eligible comparisons. The width of the lines represents the number of studies being compared, and the node size reflects the sample size. **(B)** The forest plot of network results. The black diamonds represent the combined ORs; OR > 1 indicates that the proportion of 3M-FI in the former group is greater than that in the latter group. **(C)** The cumulative ranking curve of 3M-FI. **(D)** The ranking of different doses of tPA is based on the cumulative probability plots. Ranking first means having the highest proportion of 3M-FI.

Eleven studies involving 8,151 patients reported 3M-FI (Figure 3A). The pooled results showed no significant difference between different doses of tPA (Figure 3B). The probability-based ranking result is shown in Figures 3C,D. Results of SUCRA showed that the standard dose ranked first (90.2%), the moderate dose ranked second (36.9%), and the low dose ranked third (22.9%). In this result, the first rank had the highest proportion of 3M-FI.

**Figure 4:**
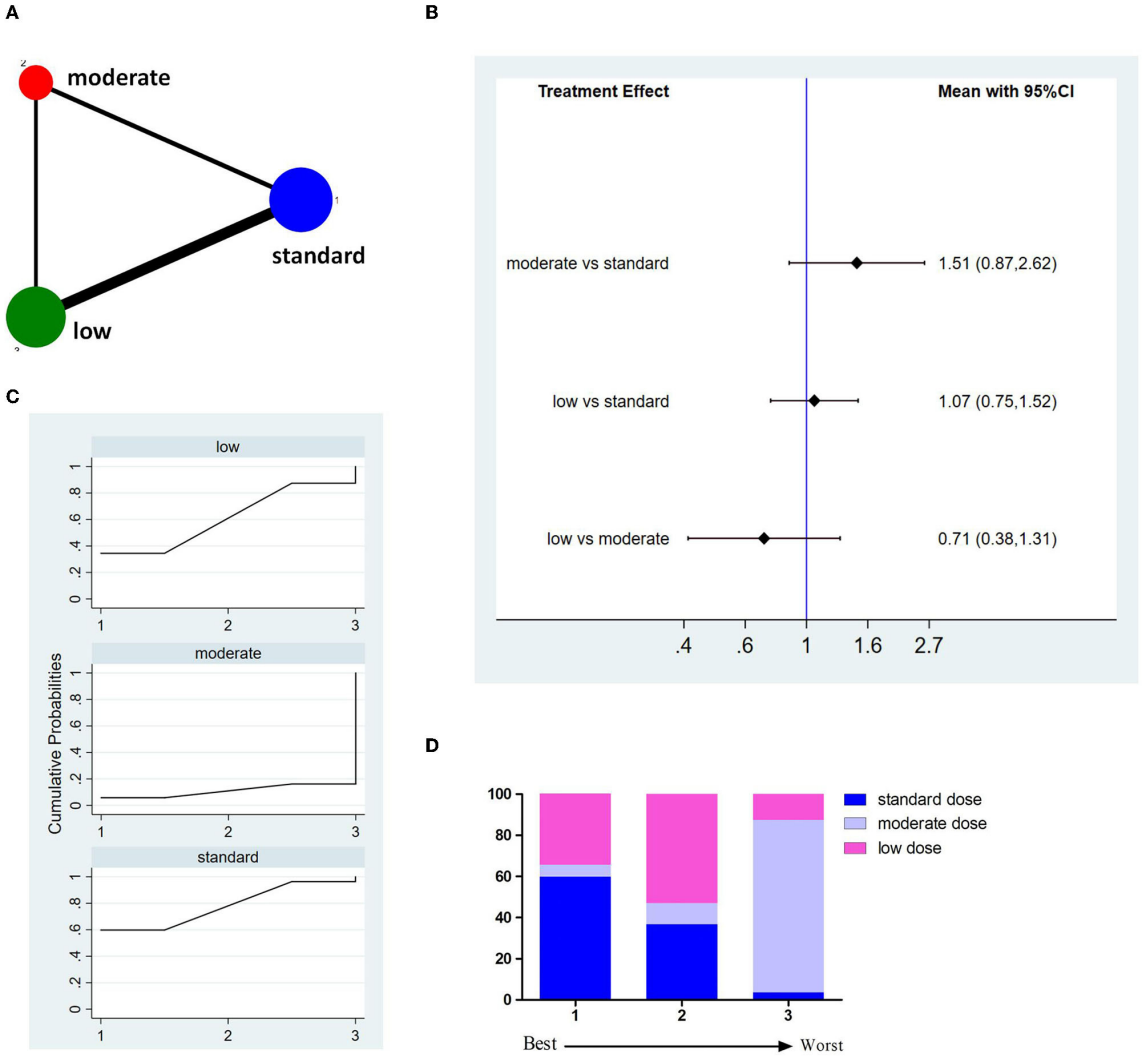
Results of symptomatic intracranial hemorrhage (sICH). **(A)** Network plots of eligible comparisons. The width of the lines represents the number of studies being compared, and the node size reflects the sample size. **(B)** The forest plot of network results. The black diamonds represent the combined ORs; OR > 1 indicates that the incidence rate of sICH in the former group is higher than in the latter group. **(C)** The cumulative ranking curve of sICH. **(D)** The ranking of different doses of tPA is based on the cumulative probability plots. Ranking first means having the lowest incidence of sICH.

Fourteen studies involving 8,614 patients reported incidences of sICH (Figure 4A). The pooled results showed no significant difference between different doses of tPA (Figure 4B). The probability-based ranking result is shown in Figures 4C,D. Results of SUCRA showed that the standard dose ranked first (78.1%), the low dose ranked second (61.0%), and the moderate dose ranked third (11.0%). In this result, the first rank had the lowest incidence of sICH.

**Figure 5:**
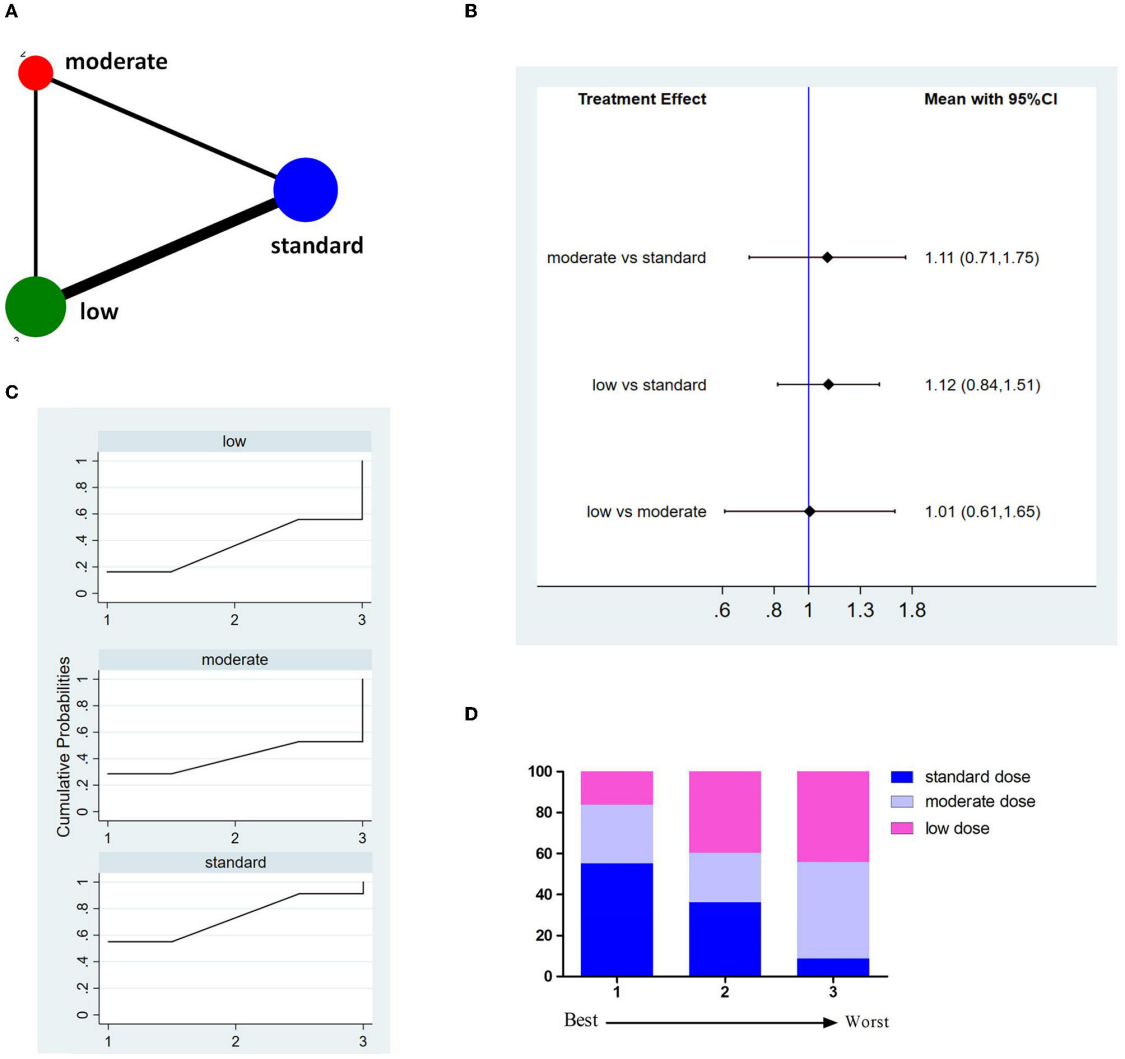
Results of 3-month all cause-mortality (3M-M). **(A)** Network plots of eligible comparisons. The width of the lines represents the number of studies being compared, and the node size reflects the sample size. **(B)** The forest plot of network results. The black diamonds represent the combined ORs; OR > 1 indicates that the incidence rate of 3M-M in the former group is higher than in the latter group. **(C)** The cumulative ranking curve of 3M-M. **(D)** The ranking of different doses of tPA is based on the cumulative probability plots. Ranking first means having the lowest incidence of 3M-M.

Thirteen studies involving 8,699 patients reported incidences of 3M-M (Figure 5A). The pooled results showed no significant difference between different doses of tPA (Figure 5B). The probability-based ranking result is shown in Figures 5C,D. Results of SUCRA showed that the standard dose ranked first (73.2%), the moderate dose ranked second (40.8%), and the low dose ranked third (36.1%). In this result, the first rank had the lowest incidence of 3M-M.

### Consistency Analysis and Publication Bias

**Figure 6:**
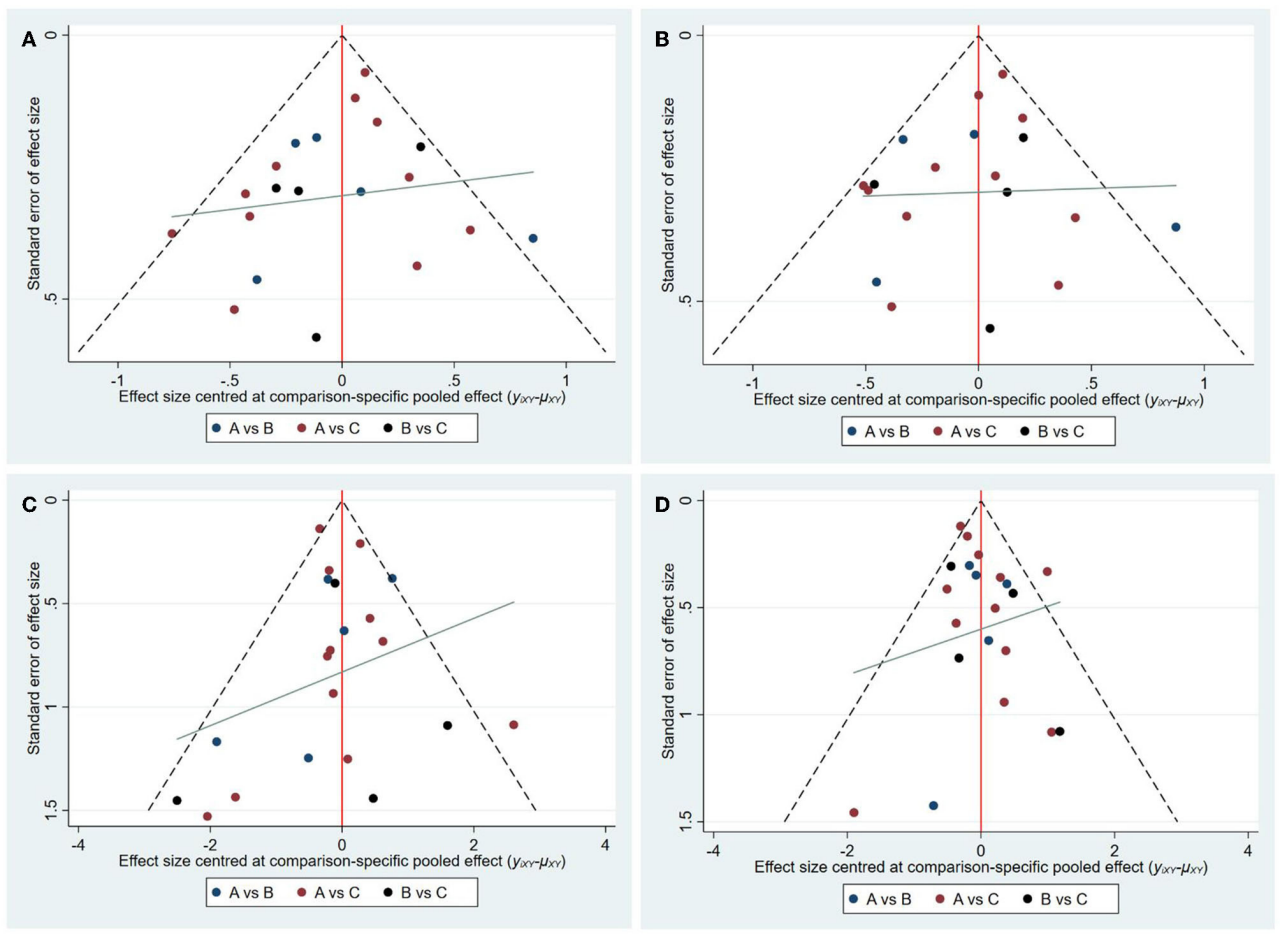
Funnel plots of each outcome. **(A)** Funnel plots of 3M-FF; **(B)** Funnel plots of 3M-FI; **(C)** Funnel plots of sICH; **(D)** Funnel plots of 3M-M. (A: standard dose, B: moderate dose, C: low dose).

The node-splitting analysis was applied to evaluate the inconsistency by comparing the differences between direct and indirect evidence. No significant inconsistency was shown indicating that the results were reliable. With regard to publication bias, no asymmetry evidence was shown in funnel plots (Figure 6).

## Discussion

To date, different doses of tPA have been applied in AIS, but it is not determined which one is the best. Traditional meta-analysis only allows the direct comparison of two doses that have been evaluated head-to-head, while NMA can compare all doses of tPA simultaneously within a single framework and rank available doses of tPA according to efficacy and safety. NMA is helpful for clinical decision-making by providing information on which is the best treatment.

After the studies (3–5) performed in Japan demonstrated that the efficacy and safety of low dose (0.6 mg/kg) tPA was comparable to the standard dose, many studies have evaluated the efficacy and safety of different doses of tPA in AIS, which varied from 0.5 mg/kg to 0.9 mg/kg. In terms of efficacy, four studies showed that the standard dose was better than lower doses (7, 9, 31, 36) and an opposite conclusion was reported in two studies (19, 30). In terms of safety, two studies showed that sICH occurred more frequently with a low dose (14.5%) (31, 36); two studies showed that there were significantly fewer sICH with a low dose of tPA (20, 39); one study showed that the 3-month mortality rate was higher in the standard dose group (30). In our study, we did not find any difference between low (<0.7 mg/kg), moderate (0.8 mg/kg), and standard (0.9 mg/kg) doses of tPA with regard to efficacy and safety.

Although there was no difference in the efficiency and safety of different doses, results of SUCRAs demonstrated that the standard dose ranked as the most effective dose. Namely, the standard dose was still the best option for patients with AIS. With respect to an individual patient, who may be suitable for a lower dose? One study including 3,479 patients with AIS reported that in patients who had moderate stroke (NIHSS 5–14), lower doses of tPA are associated with reduced sICH and non-inferior performance in efficacy (29). Another study demonstrated that a low dose (0.6 mg/kg) of tPA may be preferable in patients with AIS with younger age, lower systolic blood pressure, and mild neurological impairment (28). Moreover, in patients aged 71–80 years, there was a significant trend of increasing sICH (*P* = 0.013) and fewer good functional outcomes (*P* = 0.0179) with increasing doses of tPA (19). Therefore, future studies should observe the efficacy and safety of different doses of tPA in different patient populations with AIS as discussed above. In addition, considering the high medical burden, lower doses of tPA may be suitable for those patients with poor financial ability as it was reported that the total cost during hospitalization for the 0.6 mg/kg dose group was significantly less than that for the standard-dose group (3,401.7 USD vs. 4,157.4 USD) (38).

To better interpret the results, some limitations of this NMA should be acknowledged. First, the present study was not based on individual patient data, which limited the evaluation of other clinical outcomes and the influence of other confounders, such as the combination of characteristics as discussed above. Secondly, most of the included studies focused on the Asian population, which cannot exclude racial differences. Third, most of the studies were observational and only one was a large international RCT, which may affect the validity of our results. Fourth, baseline characteristics of included studies have not been analyzed; thus, the comparability of results between studies may be debatable.

## Conclusion

In summary, the results from this NMA suggest that lower dose tPA is comparable to the standard dose with regard to efficacy and safety. Based on the SUCRA results and AHA/ASA guidelines (2), the standard dose was still the optimal selection for AIS. Due to the limitations of the present study, further studies of high quality are needed.

## Data Availability Statement

The original contributions presented in the study are included in the article/Supplementary Material, further inquiries can be directed to the corresponding author.

## Author Contributions

B-HL, J-HW, and N-WY conceived and designed the study and were involved in literature search and data collection. B-HL, HW, and D-ZW analyzed the data. B-HL, SY, and F-QG wrote the paper. J-HW and N-WY reviewed and edited the manuscript. All authors have read and approved the final manuscript.

## Conflict of Interest

The authors declare that the research was conducted in the absence of any commercial or financial relationships that could be construed as a potential conflict of interest.

## Publisher's Note

All claims expressed in this article are solely those of the authors and do not necessarily represent those of their affiliated organizations, or those of the publisher, the editors and the reviewers. Any product that may be evaluated in this article, or claim that may be made by its manufacturer, is not guaranteed or endorsed by the publisher.

## Supplementary Material

Supplementary Figure 1 Results of the bias risk assessment.

Supplementary Table 1 Search strategy.
