# Supplementary figures and images for: Different Doses of Intravenous Tissue-Type Plasminogen Activator for Acute Ischemic Stroke: A Network Meta-Analysis
Source: Front Neurol. 2022 Jun 23;13:884267. doi: 10.3389/fneur.2022.884267 (PMC9259871; doi:10.3389/fneur.2022.884267)

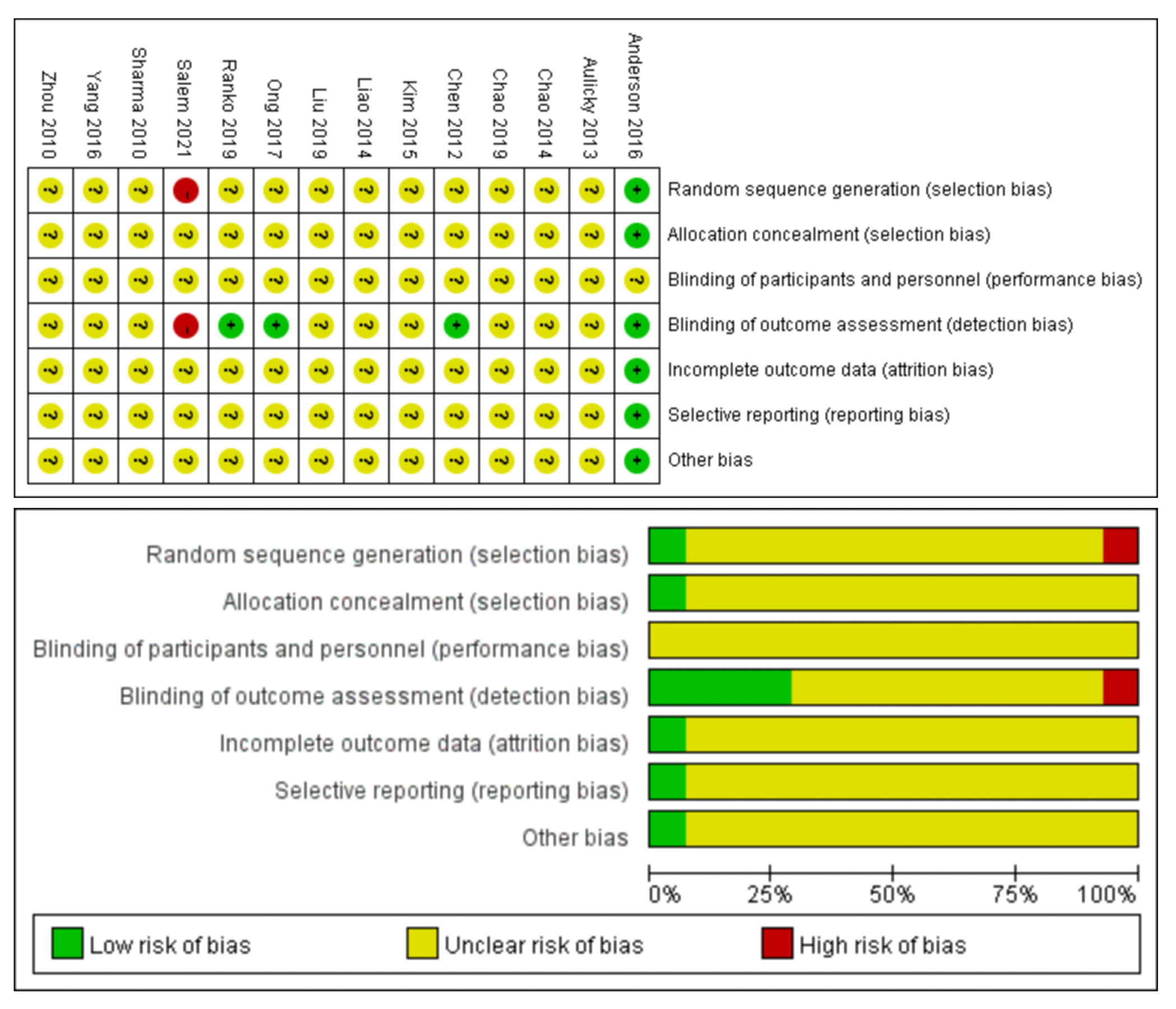

Supplement: Supplementary Figure 1 — Results of the bias risk assessment. [file Image_1.JPEG]
