# Supplementary material for: Different Doses of Intravenous Tissue-Type Plasminogen Activator for Acute Ischemic Stroke: A Network Meta-Analysis
Source: Front Neurol. 2022 Jun 23;13:884267. doi: 10.3389/fneur.2022.884267 (PMC9259871; doi:10.3389/fneur.2022.884267)
Supplement: Supplementary Table 1 — Search strategy. [file Table_1.DOCX]

**PubMed**

((low dose OR different dose) AND (alteplase OR tissue plasminogen activator OR intravenous thrombolysis OR rtPA)) AND (stroke OR cerebral infarction OR cerebral ischemia) Filters: English

**Embase**

1 exp stroke

2 stroke OR cerebral infarction OR cerebral ischemia

3 1 or 2

4 exp thrombolysis

5 alteplase OR tissue plasminogen activator OR intravenous thrombolysis OR rtPA

6 4 or 5

7 low dose OR different dose

8 3 and 6 and 7

**Web of Science**

4 #3 AND #2 AND #1

3 TS=(low dose OR different dose)

2 TS=(alteplase OR tissue plasminogen activator OR intravenous thrombolysis OR rtPA)

1 TS=(stroke OR cerebral infarction OR cerebral ischemia)
